# Crystal structure of the catalytic unit of GH 87-type α-1,3-glucanase Agl-KA from *Bacillus circulans*

**DOI:** 10.1038/s41598-019-51822-5

**Published:** 2019-10-25

**Authors:** Shigekazu Yano, Wasana Suyotha, Natsuki Oguro, Takashi Matsui, Shota Shiga, Takafumi Itoh, Takao Hibi, Yoshikazu Tanaka, Mamoru Wakayama, Koki Makabe

**Affiliations:** 1 Department of Biochemical Engineering, Graduate School of Sciences and Engineering, Yamagata University, Jonan, Yonezawa, Yamagata, 992-8510 Japan; 2 Department of Industrial Biotechnology, Faculty of Agro-industry, Prince of Songkla University, Hat Yai, 90112 Thailand; 3 Graduate School of Life Sciences, Tohoku University, Sendai, 980-8577 Japan; 4 Department of Bioscience and Biotechnology, Faculty of Biotechnology, Fukui Prefectural University, Eiheiji-cho, Yoshida-gun, Fukui, 910-1195 Japan; 5 Department of Biotechnology, Faculty of Life Sciences, Ritsumeikan University, Kusatsu, Shiga 525-8577 Japan

**Keywords:** X-ray crystallography, Glycobiology, Enzymes

## Abstract

Glycoside hydrolase (GH) 87-type α-1,3-glucanase hydrolyses the α-1,3-glucoside linkages of α-1,3-glucan, which is found in fungal cell walls and extracellular polysaccharides produced by oral *Streptococci*. In this study, we report on the molecular structure of the catalytic unit of GH 87-type α-1,3-glucanase, Agl-KA, from *Bacillus circulans*, as determined by x-ray crystallography at a resolution of 1.82 Å. The catalytic unit constitutes a complex structure of two tandemly connected domains—the N-terminal galactose-binding-like domain and the C-terminal right-handed β-helix domain. While the β-helix domain is widely found among polysaccharide-processing enzymes, complex formation with the galactose-binding-like domain was observed for the first time. Biochemical assays showed that Asp1067, Asp1090 and Asp1091 are important for catalysis, and these residues are indeed located at the putative substrate-binding cleft, which forms a closed end and explains the product specificity.

## Introduction

α-1,3-Glucanase (E.C.3.2.1.59) hydrolyses the α-1,3-glucoside linkages in α-1,3-glucan, which is found in fungal cell walls and extracellular polysaccharides produced by oral *Streptococci*^1,2^. α-1,3-Glucanases are classified into family 71 and 87 of glycoside hydrolases (GHs) based on their amino acid sequences. GH 71-type α-1,3-glucanases are found in fungi^3–6^, while GH 87-type enzymes are found in bacteria^7–9^. GH 87-type α-1,3-glucanase was isolated from the culture filtrate of *Bacillus circulans* KA-304 and termed Agl-KA. Agl-KA hydrolyses the α-1,3-glucan of oral *Streptococci* as well as of fungi^10^. Accordingly, Agl-KA has been investigated for preparing fungal protoplasts, for the analysis of α-1,3-glucan in fungal cell walls and for the biological control of pathogenic fungi^11,12^.

**Figure 1:**
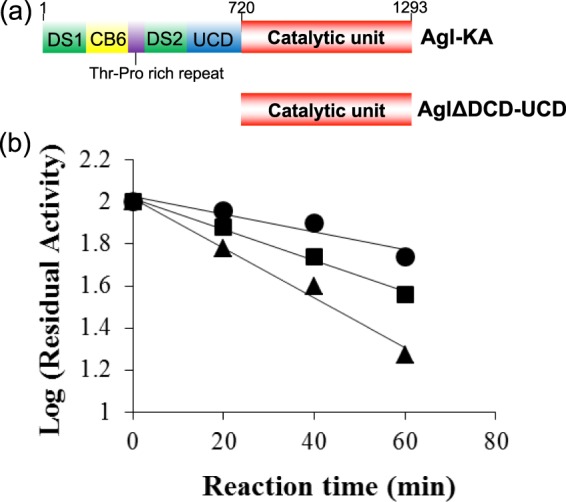
(**a**) Domain structure of Agl-KA. DS: Discoidin domain (green), CB6: Carbohydrate-binding module 6 (yellow), UCD: Uncharacterised domain (blue). AglΔDCD-UCD: the catalytic unit of Agl-KA (red). (**b**) Residual activity of AglΔDCD-UCD after chemical modification with the concentrations of 25 mM (●), 50 mM (■) and 100 mM (▲) EDC.

Most GH 87 enzymes are considered multidomain enzymes. Agl-KA is also multimodular, comprising an N-terminal discoidin domain (DS1), followed by a carbohydrate-binding module 6 (CB6), threonine and proline repeats (TP), a second discoidin domain (DS2), an uncharacterised conserved domain (UCD) and a C-terminal catalytic unit (AglΔDCD-UCD; Fig. 1a)^13^. The comparison of amino acid sequences of GH 87 enzymes suggests that these domains are conserved in GH 87-type α-1,3-glucanases. In our previous study, we found that DS1, CB6 and DS2 are involved in α-1,3-glucan binding and that these domains enhance the α-1,3-glucan hydrolysing activity of the catalytic unit^14^. Furthermore, we reported that the deletion of UCD from Agl-KA does not affect α-1,3-glucan binding activity, α-1,3-glucan hydrolysing activity or fungal cell wall lytic activity. While these findings have revealed the functions of the individual domains of GH 87 α-1,3-glucanase, the molecular enzymatic mechanisms based on the three-dimensional structure remain unknown.

In this study, we successfully determined the x-ray crystal structure of AglΔDCD-UCD using the Se-MAD method. We further discussed the product specificity using a combination of biochemical analysis and docking simulation.

## Results and Discussion

### Chemical modification of carboxyl groups in the catalytic unit of Agl-KA

To reveal amino acids essential for α-1,3-glucan hydrolysis, AglΔDCD-UCD—the catalytic unit of Agl-KA—was treated with 1-ethyl-3-(3-dimethyl amino propyl) carbodimide (EDC), a chemical modifier of carboxylic amino acids used for the inhibition of enzymatic reactions. Enzyme hydrolysis decreased with incubation time and EDC concentration; after incubation for 60 min with 100 mM EDC, the activity was approximately 18% of the initial activity (Fig. 1b; 10^1.25^ = 18%). These results suggest that at least one carboxylic amino acid residue is involved in catalysis and/or the substrate binding of α-1,3-glucanase.

### Mutational analysis of key residues for catalysis

Multiple alignments of the amino acid sequences of the catalytic unit from several GH 87 α-1,3-glucanases indicated that 11 carboxylic residues at positions equivalent to D737, D739, E763, D853, E854, D884, E889, E1032, D1067, D1090 and D1091 of Agl-KA were conserved among α-1,3-glucanases (Supplementary Fig. S1). These 11 carboxylic amino acid residues were substituted with alanine to further clarify the amino acids involved in Agl-KA catalysis.

Mutant enzymes were expressed in *E. coli*, and resultant mutant proteins were purified from the soluble fraction of *E. coli* according to the purification method for wild-type AglΔDCD-UCD^13^. Final purity was confirmed using SDS–PAGE (Supplementary Fig. S2). The relative activities of the wild-type and mutant enzymes are shown in Table 1. The mutants of D737A, D739A, D853A, E854A and E1032A retained approximately 80%–100% of their wild-type activity, indicating that the substitution of these residues had little effect on α-1,3-glucanase activity. Activities of E763A and E889A considerably decreased; however, they remained at approximately 30%–40% of their wild-type activity. In contrast, the activities of D1067A, D1090A and D1091A were not detected after 30 min of incubation. After 18 h of incubation, D1067A, D1090A and D1091A showed only 0.09%, 2.84% and 3.12% of their wild-type activity, respectively. These findings suggest that D1067, D1090 and D1091 are important for Agl-KA catalysis.

**Table 1:** α-1,3-Glucanase activity of mutant enzymes.

Mutation sites	Relative activity (%)	
	Reaction time 30 min	Reaction time 18 h
Wild-type (AglΔDCD-UCD)	100.0	100.0
D737A	88.8	109.2
D739A	100.3	106.2
E763A	28.8	45.3
D853A	80.8	86.9
E854A	92.4	98.8
D884A	80.4	79.5
E889A	39.4	63.1
E1032A	96.3	85.3
D1067A	n.d.	0.09
D1090A	n.d.	2.84
D1091A	n.d.	3.12
D1067E	4.36	41.0
D1090E	5.99	36.7
D1091E	21.9	45.4
D1067N	n.d.	1.96
D1090N	n.d.	0.12
D1091N	n.d.	0.07

Subsequently, D1067, D1090 and D1091 were individually substituted with asparagine or glutamic acid. The activities of D1067N, D1090N and D1091N were only hardly detectable even after the extended incubation for 18 h (Table 1). In the glutamic acid substitution mutants, the activities of D1067E, D1090E and D1091E were 4.36%, 5.99% and 21.89% of their wild-type activity after 30 min incubation, respectively. The kinetic parameters of D1067E, D1090E, D1091E and wild-type were obtained from Lineweaver–Burk plots (Supplementary Table S2). The *K*_m_ values for D1067E, D1090E, D1091E and wild-type were approximately 42.1, 35.4, 72.4 and 36.2 mg/mL, respectively. The turnover numbers (*k*_cat_) of D1067E, D1090E, D1091E and wild-type were approximately 6.1, 6.4, 49.7 and 81.5 sec^−1^, respectively. The *k*_cat_ values of D1067E and D1090E were approximately 13.5-fold lower than that of wild-type; however, the apparent *K*_m_ values were similar to that of wild-type. In contrast, the *k*_cat_ value of D1091E was approximately 2-fold lower and the *K*_m_ value was approximately 2-fold higher than that of wild-type. The results of kinetic analysis indicate that D1067 and D1090 may be involved in Agl-KA catalysis and D1091 may be important for substrate binding.

**Figure 2:**
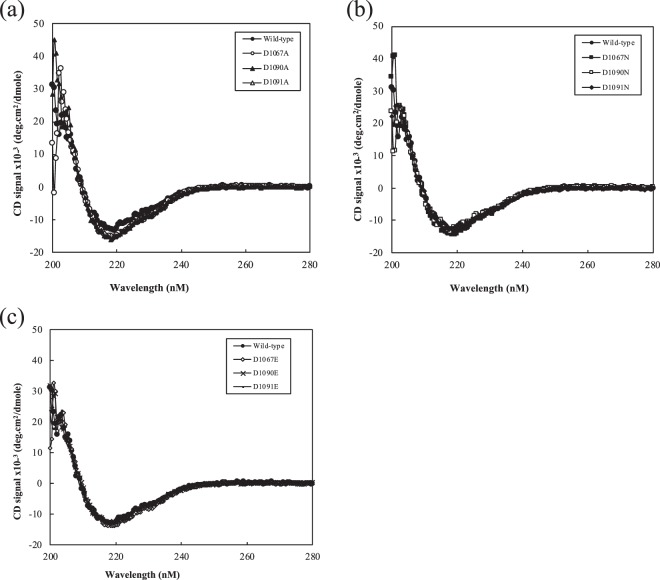
CD spectra of Agl∆DCD-UCD with alanine (**a**), asparagine (**b**) and glutamic acid (**c**) substitution mutants. Abbreviations: ●, wild-type: ○, D1067A: ▲, D1090A: ∆, D1091A: ■, D1067N: □, D1090N: ♦, D1901N, ◊, D1067E: x, D1090E: —, D1091E.

Next, far-UV circular dichroism (CD) spectra of the D1067, D1090 and D1091 substituted mutants were obtained to confirm whether the overall secondary structures of enzymes were maintained. Figure 2 shows the CD spectra of the mutants and wild-type measured at the far-UV range (200–280 nm). All mutants showed CD spectra similar to that of wild-type, indicating that amino acid substitution did not significantly affect their secondary structure.

### Crystal structure of AglΔDCD-UCD

**Figure 3:**
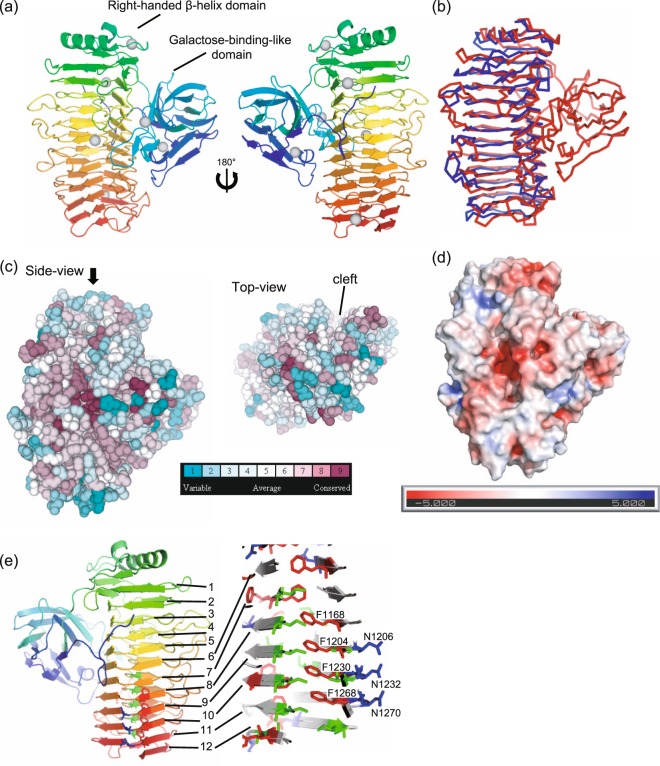
Crystal structure of AglΔDCD-UCD (PDBID: 5ZRU). (**a**) Ribbon representation of the structure coloured with the amino acid sequence from the N- (blue) to C-terminals (red). Six Ca^2+^ ions are shown as grey spheres. (**b**) Structure comparison with a similar structure protein, a catalytic A-module of a mannuronan C-5-epimerase (AlgE4A) structure (PDBID: 2PYH; blue), from Dali server is shown with Agl∆DCD-UCD (red). (**c**) Amino acid conservation is shown with colour as indicated. The direction of the top view is shown with a black arrow. A putative substrate-binding cleft is indicated by the top view. (**d**) Electrostatic potential of the molecular surface of AglΔDCD-UCD obtained using the APBS tool. The negative potential is shown in red, and the positive potential is shown in blue. (**e**) The twelve β-helix coils. The phenylalanine (red), asparagine (blue) and isoleucine (green) ladders are shown with stick representation.

The crystal structure of AglΔDCD-UCD was determined at a resolution limit of 1.83 Å (Fig. 3). The crystal belonged to the space group of P2_1_, and the structure was refined to the R-values of 14.1% and 17.1% for R and R_free_, respectively (Table 2). Two molecules (molecules A and B) were positioned in the asymmetric unit and were essentially identical (root mean square deviation, 0.14 Å); hereafter, molecule A is used for discussion. All 575 amino acids of AglΔDCD-UCD were clearly resolved in the crystal structure, with the exception of the cloning artefact of the N-terminus Met–Ser sequence. Five Ca^+^ ions, two Zn^2+^ ions, two SO_4_2^−^ molecules and one polyethylene glycol chain were observed within one protein molecule. Zn^2+^ ions, SO_4_^2−^ molecules and polyethylene glycol were contained in the crystallisation buffer, and these molecules were localised to the protein surface; thus, we concluded that these were introduced during crystallisation. All Zn^2+^ ions were positioned at the interfaces of the protein molecules, contributing to the crystal packing. The positions of three Ca^+^ ions (out of five) were relatively buried in the protein structure, indicating these may contribute to protein stability (Fig. 3a). Consistent with this, inductively coupled plasma-mass spectrometry (ICP–MS) showed that nearly 40 nmol Ca^2+^ ions per 10 nmol protein molecules were contained in purified AglΔDCD-UCD solution, whereas no Zn^2+^ ions were detected.

**Table 2:** Data collection and refinement statistics for AglΔDCD-UCD (PDBID: 5ZRU).

Space group	Native	SeMet		
	P2_1_	P2_1_		
***Data collection statistics***				
Cell parameters	a = 57.25			
	b = 126.42			
	c = 80.54			
	γ = 97.83			
**Beamline**	**PF-BL5A**	**PF-BL5A**		
		**Peak**	**Edge**	**Remote**
Wavelength(Å)	1.0000	0.97911	0.97922	0.9640
Resolution (Å)^a)^	20.0–1.83 (1.86-1.83)	49.7–2.00 (2.12-2.00)		
Completeness (%)	99.4 (95.5)	99.8 (99.3)	99.5 (98.5)	99.5 (98.5)
I/σ (I)	13.29 (3.00)	8.15 (2.24)	5.46 (1.36)	5.30 (1.35)
R_merge_^b)^	0.050(0.347)	0.206 (0.856)	0.204 (0.886)	0.212 (0.897)
Average redundancy	3.4 (3.4)	7.0 (7.0)	3.49 (3.47)	3.49 (3.48)
**Refinement statistics**				
Resolution range (Å)	19.9–1.83			
Reflections used (free)	98636 (4962)			
R factor ^c)^	0.141			
R_free_^d)^	0.171			
*RMS deviations*				
Bonds (Å)	0.006			
Angles (°)	0.846			
Average B factor (Å^2^)	15.0			
***Ramachandran plot statistics***				
Most favoured (%)	96			
Generously allowed (%)	4			
Disallowed (%)	0			

^a)^The highest resolution shell is shown in parenthesis. ^b)^R merge = Σ_hkl_Σ_i_|I(hkl)_i_ − 〈I(hkl)〉|/Σ_hkl_Σ_i_〈I(hkl)_I_〉 over *i* observations of a reflection hkl. ^c)^R factor = Σ||F(obs)| − |F(calc)||/Σ|F(obs)|. ^d)^Rfree is R with 5% of reflections sequestered before refinement.

The overall structure of AglΔDCD-UCD comprised an N-terminal galactose-binding-like domain (~180 residues), followed by a right-handed β-helix (~400 residues), as determined based on the CATH domain classification^15^. This is consistent with the prediction by the InterPro protein sequence classification server^16^ (Fig. 3a). According to the structural alignment search server, Dali, the catalytic A-module of the mannuronan C-5-epimerase (AlgE4A) structure (PDBID: 2PYH), showed the highest Z-score of 37.4 and a sequence homology of 18%. Though the AlgE4A structure appeared to have a similar right-handed β-helix fold, it lacked the galactose-binding-like domain, highlighting the unique entire structure of AglΔDCD-UCD (Fig. 3b). In addition to the two domains, there appeared to be an N-terminal extension before the galactose-binding-like domain (~14 residues) as well as a linker between the two domains (~11 residues). These regions were resolved with clear electron density maps (Supplementary Fig. S3a,b). The entire structure of AglΔDCD-UCD was β-sheet rich and consistent with the observed CD spectra (Fig. 2a). The right-handed β-helix fold is a common structural class for carbohydrate-binding proteins called “CASH (carbohydrate-binding proteins and sugar hydrolase)”^17^, and the C-terminal catalytic unit of the GH 87 family was also predicted to share this common fold^18^.

The striking difference between AglΔDCD-UCD and other β-helix folds of CASH is the presence of the galactose-binding-like domain, which forms a complex with the β-helix domain (Fig. 3a). The interface area between the galactose-binding-like domain and the β-helix domain was 1743 Å2, indicating that a large surface area was buried upon complex formation and the complex was stabilised. The amino acid sequence of the galactose-binding-like domain is highly conserved among the GH 87 family (Supplementary Fig. S1), indicating that this complex structure is a common structural feature in this family. Figure 3c shows the conserved amino acids in the GH 87 family; highly conserved residues are located at the bottom of the cleft, which is a putative active site. Amino acids at the interface between the galactose-binding-like domain and the β-helix domain are also conserved (Supplementary Fig. S3c). Figure 3d shows the electrostatic surface potential and putative active site at the conserved substrate-binding cleft with negative surface potential.

Although the structure of Dex49, a GH 49 family dextranase, shows a similar complex structure comprising an N-terminal β-sandwich domain and a β-helix domain^19^, the β-sandwich domain shares no sequence homology with the galactose-binding-like domain and the secondary structure topology is also different. Despite the proposal that the GH 49 and GH 87 families share a common evolutionary ancestor^18^, these complex structures seem to be independently acquired during evolution.

The β-helix consists of 12 turns, with 24–38 amino acids per turn (Fig. 3e). According to the original definition of a β-helix by Yoder *et al*., one β-helix consists of three β-strands, PB1, PB2 and PB3, connected via three turns, T1, T2 and T3^20^. The N- and C-terminal coils are considered incomplete coils comprising PB2 and PB3 for the N-terminal coil and PB1 and PB2 for the C-terminal coil. This is commonly observed as inter-strand aromatic stacking and an asparagine ladder in the β-helix fold^20^; such ladders are observed at the C-terminal β-helix of AglΔDCD-UCD (Fig. 3c). Four phenylalanine residues (F1168, F1204, F1230 and F1268) are stacked inside the PB2, and three asparagine residues (N1208, N1232 and N1270) are aligned at the coils of 8, 9, 10 and 11. Isoleucine residues are also aligned at PB1, which is at the opposite side of the phenylalanine ladder. The β-helix fold creates a concave surface along with the β-helix direction, resulting in a consecutive groove of the putative substrate recognition cleft (Fig. 3c,d).

**Figure 4:**
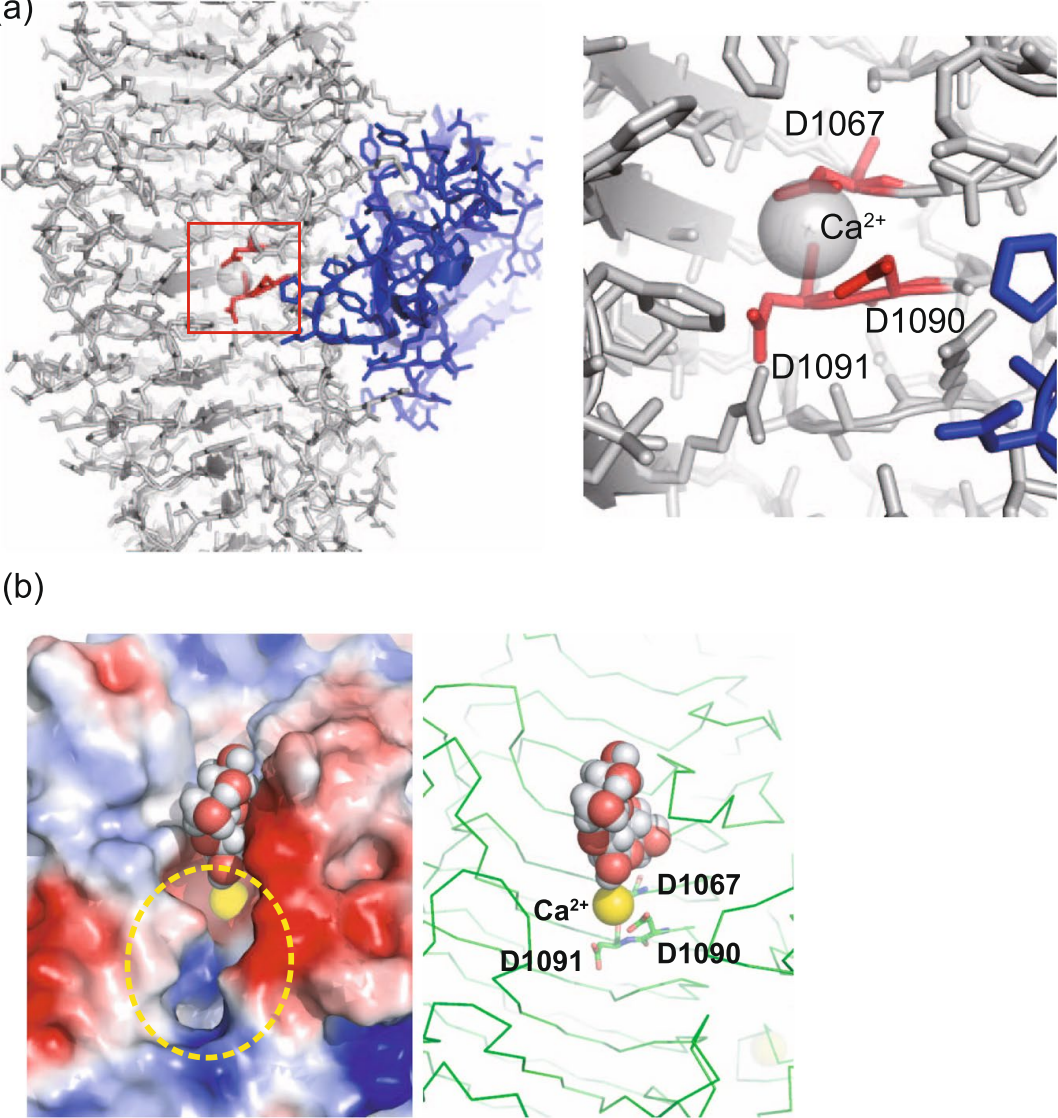
(**a**) Positions of D1067, D1090 and D1091. These three residues are shown in red. The galactose-binding-like domain is shown in blue. The enlarged view is shown at right. (**b**) Docking simulation results using a nigerose molecule. Left: the nigerose molecule is shown with sphere. Right: the glutamate triad is shown with stick representation, and Ca^2+^ is shown with a yellow sphere. Protein Cα trace is shown with green line. Substrate-binding pocket is indicated with a dashed yellow circle.

The crystal structure revealed the positions of mutational residues that affected enzymatic activity. Mutations of D763, E889, D1067, D1090 and D1091 led to <50% enzyme activity after 30 min of incubation. Among these residues, D763 and E889 were located in the galactose-binding-like domain at the interface with the β-helix domain; these are not likely to be involved in the enzymatic reaction directly because these are locate relatively far from the putative substrate-binding pocket (Supplementary Fig. S4). Rather, these positions may be important for complex formation via domain–domain interactions. In particular, E889 forms an ionic pair with R1060 of the β-helix domain (Supplementary Fig. S4). Thus, mutations in these two residues may result in the destabilisation of the complex. Mutations of D1067, D1090 and D1091 drastically reduced enzymatic activity, particularly with alanine and asparagine substitutions (Table 1). Interestingly, these three aspartate residues are conserved among the GH 28^21–23^ and GH 49^19^ families and appear to form the active site. Based on sequence similarity, these residues were predicted to also serve as the putative catalytic site for the GH 87 family^18^. These three residues are located at the very centre of the putative active cleft (Fig. 4a). Interestingly, a Ca^2+^ ion is coordinated to the carboxy group of D1067, D1090 and water molecules, with pentagonal–bipyramidal coordination. A similar Ca^2+^ ion coordination on the β-helix was observed in polysaccharide lyase, contributing to polysaccharide binding and enzymatic activity^24,25^. Thus, Ca^2+^ at the catalytic site of AglΔDCD-UCD may have a similar role in the reaction.

At the centre of the substrate-binding cleft, a loop from the galactose-binding-like domain appears to be protruding close to the D1067, D1090 and D1091 sites (Fig. 4a). Although the role of the loop of the galactose-binding-like domain remains unclear, this interesting structural feature may contribute to α-1,3-glucane recognition via its saccharide-binding ability.

### Docking simulation using nigerose

To evaluate the substrate binding of AglΔDCD-UCD, we performed docking simulation using a nigerose molecule, a disaccharide molecule with two glucose molecules linked via a α-1,3 linkage as the ligand. One of the solutions showed positioning at the substrate-binding cleft near D1067, D1090 and D1091 (Fig. 4b). Although docking is a rough estimation of the binding mode, it at least represents a possible binding structure. The putative-1 subsite is located onto the carboxy group of D1067. According to the reaction mechanism of the GH 28 and GH 49 families, the cleavage of the α-1,3 linkage may occur by the inverting mechanism with acid–base catalysis at this position.

A binding pocket (dashed yellow circle, Fig. 4b) is located on the opposite side of the docked nigerose molecule, with the size of a dimer-to-tetramer saccharide molecule. Thus, it is feasible to hydrolyse the α-1,3 covalent bond of α-1,3-glucan at the dimer-to-tetramer length. Indeed, Agl-KA dominantly produces tetrasaccharide, and disaccharide is also released^26^. This structure strikingly explains the molecular basis of the enzyme reaction. We hypothesise that the binding subsites are (−2)(−1)(+1)(+2)(+3)(+4) and propose a reaction mechanism as follows: (I) α-1,3-glucan binds to the binding cleft; (II) the α-1,3-glucan is hydrolysed and a nick is formed; (III) the processed α-1,3-glucan is translocated to the end of the pocket and is hydrolysed; and (IV) the tetrasaccharide is released from the pocket.

In a previous study, we have reported the x-ray crystallographic analysis of the catalytic unit of α-1,3-glucananse AglFH1 from *Paenibacillus glycanilyticus* FH11 (approximately 20% identity with AglΔDCD-UCD), which was prepared using a *Brevibacillus* expression system^27^. The crystal structure of the catalytic unit of AglFH1 was determined by the Native-SAD method, and crystallographic analysis of the complexes of AglFH1 with dimeric, trimeric or tetrameric saccharides of α-1,3-glucan are currently underway. These results will provide information on the substrate-binding pocket and insights into the hydrolysing mechanism of α-1,3-glucan based on comparison with the results of AglΔDCD-UCD.

## Conclusions

Here, we describe the novel structural feature of the C-terminal catalytic unit of GH 87-type α-1,3-glucanase from *B. circulans* KA-304. The enzyme structure explains the molecular mechanism of the reaction product because of the size of the reaction pocket. Because of the scarcity of α-1,3-glucan, the biochemical analysis of α-1,3-glucanase represents potential difficulty. In this regard, structural analysis plays an important role for complementing the limit of the biochemical assay. The accumulating structural information of the GH 87 family enzymes, in addition to the knowledge of the substrate complex structure, should reveal the precise molecular mechanism of the enzymatic reaction in the future. Detailed understanding of the GH 87-type α-1,3-glucanase will also expand the industrial applications of this enzyme, such as in antifungal drugs, by enabling its structure-based engineering.

## Materials and Methods

### Microorganisms and culture

*E. coli* DH 5α cells were used as a host to construct various recombinant plasmids grown at 37 °C while shaking (100 rpm) in LB medium containing 100 μg/mL ampicillin. *E. coli* Rosetta-gami B (DE3), which harboured a recombinant plasmid, was grown at 30 °C while shaking (100 rpm) in LB medium containing 100 μg/mL ampicillin, 10 μg/mL chloramphenicol, 25 μg/mL kanamycin and 15 μg/mL tetracycline.

### Site-direct mutagenesis

The previously constructed pET-AglΔDCD-UCD plasmid was used as a template to generate mutants of the catalytic unit of Agl-KA. All mutant plasmids were generated using QuikChange methods (Agilent Technologies)^28^. PCR was performed under the following conditions: one cycle of 94 °C for 2 min, followed by 18 cycles of 98 °C for 10 s, 55 °C for 5 s and 72 °C for 7.5 min. Sequences of the mutagenic primers are listed in Supplementary Table S1. PCR products were treated with *Dpn*I at 37 °C for 1 h to digest the methylated template and then transformed into *E. coli* JM 109. Mutant plasmids were collected and sequenced to confirm the desired mutation.

### Enzyme production and purification

All mutant plasmids were transformed into *E. coli* Rosetta-gami B (DE3) for expression. The transformants were cultured at 30 °C in LB medium. When the optical density at 600 nm reached ~0.6, isopropyl-β-D-thiogalactopyranoside was added to the culture medium at a final concentration of 0.4 mM. Cultures were incubated further for 12 h. *E. coli* cells harbouring expression plasmids were harvested and disrupted by sonication (10 min, 350–400 µA) on ice. AglΔDCD-UCD and its mutants were purified according to the method previously described^13^.

Concentrations of AglΔDCD-UCD and mutants were estimated by measuring absorbance at 280 nm with the molar absorption coefficients (154.130 M^−1^ cm^−1^), calculated on the basis of their amino acid compositions^29^. SDS–PAGE was performed using the method of Laemmli^30^. Pre-stained Protein Markers Broad Range (Nacalai Tesque, Kyoto, Japan) was used as a molecular marker.

### α-1,3-Glucanase activity assay

The reaction solution containing 1% α-1,3-glucan, 50 mM potassium phosphate buffer (pH 6.5), and appropriate enzyme concentrations was incubated at 30 °C. The reaction was quenched by placing the sample at 100 °C for 15 min. The suspension was centrifuged, and the precipitated α-1,3-glucan was removed. The amount of the reducing sugars in the supernatant was determined using dinitrosalicylic acid according to the method of Miller^31^.

### Chemical modification of carboxyl groups

The reaction mixture containing 3 nmol/mL of AglΔDCD-UCD, 50 mM MES/NaOH (pH 5.5) and various concentrations of EDC was incubated at 25 °C. After incubation for a given period (20, 40 and 60 min), 10 μL of the reaction mixture was withdrawn and added to 40 μL of 100 mM MES/NaOH (pH 5.5) to quench the residual reagent. The residual activity of the diluted reaction mixture was then determined.

### CD measurement

The CD spectra of the purified wild-type and mutant enzymes (0.05 mg/mL) were measured at 25 °C using a spectropolarimeter (JASCO model J-820, cell light 1-cm) in the far-UV region (200–280 nm). Background was corrected against 10 mM potassium phosphate buffer (pH 6.5). The other conditions of the CD spectra were as follows: data interval, 0.5 nm; scan speed, 100 nm/min; accumulation times, 3; band width, 1.0 nm and sensitivity, 100 mdeg.

### ICP–MS analysis for Ca^2+^ and Zn^2+^ detection

ICP–MS was used for metal-ion identification. The purified enzyme was dialysed against deionised water and subsequently treated with 0.1 M HNO_3._ Samples were analysed in triplicate runs on an ICP–MS system (ELAN DRC II, Perkin Elmer Co.). Total Ca^2+^ and Zn^2+^ concentrations were measured using an external calibration curve determined with reference standards for each ion.

### Crystal structure determination

The selenomethionine derivative of Agl-KA-cat was expressed in the B834(DE3) strain using M9 medium with 0.0025% selenomethionine and 0.04% of an amino acid mix containing lysine, leucine, isoleucine, threonine, phenylalanine and valine. Native and selenomethionine derivative crystals were obtained using the hanging-drop vapour diffusion method with crystallisation buffers containing 10%–13% PEG6000, 10 mM ZnSO_4_ and 0.1 M HEPES pH 8.5 at 20 °C. The obtained crystals were then soaked in the crystallisation buffer with 30% PEG400 as a cryoprotectant and flash cooled in liquid nitrogen. Crystals were stored until the diffraction measurement.

Synchrotron x-ray diffraction measurements were performed at the beamline BL5A of Photon Factory, Tsukuba, Japan. Crystals belonged to the space group P2_1_. MAD data were collected at the absorption peak (0.97911 Å), edge (0.97922 Å) and remote peak (0.96400 Å). The MAD and native datasets were collected at resolutions 2.0 Å and 1.83 Å, respectively. Data were indexed and integrated using the xds^32^ programmes. The initial phase was determined by the Se-MAD method with the programme Phenix.autosol^33^. The initial protein model determined by the SeMet MAD phasing was used; further refinement and model building were performed using the native dataset in Phenix.refine^33^ and coot^34^. Data collection and refinement statistics are shown in Table 2. The structural data were deposited to protein data bank through PDBj (https://pdbj.org); the assigned PDBID is 5ZRU. Molecular structures were depicted with PyMol (https://pymol.org/). Electrostatic potential was calculated using the APBS tool (Adaptive Poisson-Boltzmann Solver). Conserved amino acids in the structure were depicted using Consurf^35^. Interface calculations were performed using PDBePISA (http://www.ebi.ac.uk/pdbe/prot_int/pistart.html)^36^.

### Docking simulation

Docking simulation was performed using the SwissDock server^37^. The crystal structure of AglΔDCD-UCD was used as the target molecule and nigerose was used as the ligand. The molecular structure of nigerose was obtained from PubChem (https://pubchem.ncbi.nlm.nih.gov). Results were processed using UCSF Chimera^38^. From the docking results, the result with the highest score and nearest positions to D1067A, D1090A and D1091A in the putative binding cleft were selected.

### Preparation of α-1,3-glucan

α-1,3-Glucan was prepared from sucrose using glucosyltransferase I (GTF-I) of *Streptococcus mutans* ATCC700610 as described previously^13^. The GTF-I-expressing plasmid was (pET-gtf1) was introduced into *E. coli* Rosetta-gami B (DE3). The cell free extract of *E. coli* cells from 5 L culture was used as GTF-I preparation. The GTF-I preparation and 20% sucrose were incubated in 5 L 50 mM potassium phosphate buffer (pH 7) at 30 °C. After 48 h incubation, insoluble glucans were collected by centrifugation, and the precipitate was dissolved in 500 mL 1 M NaOH. The mixture was heated at 60 °C for 20 min, and the mixture was neutralised with 6 M HCl. The neutralised mixture was added to 500 mL cold ethanol. After centrifugation, alcohol-precipitated glucan was washed twice with distilled water and lyophilised. The lyophilised powder was used as α-1,3-glucan.

## Supplementary information

Supplementary info
