# Supplementary material for: Crystal structure of the catalytic unit of GH 87-type α-1,3-glucanase Agl-KA from Bacillus circulans
Source: Sci Rep. 2019 Oct 25;9:15295. doi: 10.1038/s41598-019-51822-5 (PMC6814745; doi:10.1038/s41598-019-51822-5)
Supplement: Supplementary file 1 — Supplementary info [file 41598_2019_51822_MOESM1_ESM.docx]

**Crystal structure of the catalytic unit of GH 87 type α-1,3-glucanase Agl-KA from *Bacillus circulans***

**Shigekazu Yano^1^, Wasana Suyotha^2^, Natsuki Oguro^1^, Takashi Matsui^3^, Shota Shiga^1^, Takafumi Itoh^4^, Takao Hibi^4^, Yoshikazu Tanaka^3^, Mamoru Wakayama^5^ and Koki Makabe^1*^**

**^1^**Department of Biochemical Engineering, Graduate School of Sciences and Engineering, Yamagata University, Jonan, Yonezawa, Yamagata 992-8510, Japan

^2^Department of Industrial Biotechnology, Faculty of Agro-industry, Prince of Songkla University, Hat Yai 90112, Thailand

^3^Graduate School of Life Sciences, Tohoku University, Sendai, 980-8577, Japan

^4^Department of Bioscience and Biotechnology, Faculty of Biotechnology, Fukui Prefectural University, Eiheiji-cho, Yoshida-gun, Fukui 910-1195, Japan

^5^Department of Biotechnology, Faculty of Life Sciences, Ritsumeikan University, Kusatsu, Shiga 525-8577, Japan

*corresponding. makabe@yz.yamagata-u.ac.jp

Supplementary Table S1 Primers for construction of AglΔDCD-UCD mutants

Primer name Oligonucleotide Sequence

D737A (F) 5-TATACCCGCTACGCGACGGATGACGCT-3

D737A (R) 5-AGCGTCATCCGTCGCGTAGCGGGTATA-3

D739A (F) 5-CGCTACGATACGGCGGACGCTGCAAGA-3

D739A (R) 5-TCTTGCAGCGTCCGCCGTATCGTAGCG-3

E763A (F) 5-CTGACTGCTTCGGCGGCATCCGGACAG-3

E763A (R) 5-CTGTCCGGATGCCGCCGAAGCAGTCAG-3

D853A (F) 5-CTCTTCCGCTTCGCGGAAGTGCACTGG-5

D853A (R) 5-CCAGTGCACTTCCGCGAAGCGGAAGAG-3

E854A (F) 5-TTCCGCTTCGACGCGGTGCACTGGAAG-3

E854A (R) 5-CTTCCAGTGCACCGCGTCGAAGCGGAA-5

D884A (F) 5-GAATATGGAGTAGCGTTCCTAGAGATT-3

D884A (R) 5-AATCTCTAGGAACGCTACTCCATATTC-3

D889A (F) 5-GACTTCCTAGAGATTGCGGCTGTGCCTGCC-3

D889A (R) 5-GGCAGGCACAGCCGCAATCTCTAGGAAGTC-3

E1032A (F) 5-AATGTATGGGTGGCGCATTTCGAATGCGGC-3

E1032A (R) 5-GCCGCATTCGAAATGCGCCACCCATACATT-3

D1067A (F) 5-AACAACCTTGCAGCGGGTGTCAACTTC-3

D1067A (R) 5-GAAGTTGACACCCGCTGCAAGGTTGTT-3

D1090A (F) 5-CGCAATAACGGCGCGGACGGCTTGGCT-3

D1090A (R) 5-AGCCAAGCCGTCCGCGCCGTTATTGCG-3

D1091A (F) 5-AATAACGGCGACGCGGGCTTGGCTGTC-3

D1091A (R) 5-GACAGCCAAGCCCGCGTCGCCGTTATT-3

D1067N (F) 5-AACAACCTTGCAAACGGTGTCAACTTC-3

D1067N (R) 5-GAAGTTGACACCGTTTGCAAGGTTGTT-3

D1090N (F) 5-CGCAATAACGGCAACGACGGCTTGGCT-3

D1090N (R) 5-AGCCAAGCCGTCGTTGCCGTTATTGCG-3

D1091N (F) 5-AATAACGGCGACAACGGCTTGGCTGTC-3

D1091N (R) 5-GACAGCCAAGCCGTTGTCGCCGTTATT-3

D1067E (F) 5-AACAACCTTGCAGAAGGTGTCAACTTC-3

D1067E (R) 5-GAAGTTGACACCTTCTGCAAGGTTGTT-3

D1090E (F) 5-CGCAATAACGGCGAAGACGGCTTGGCT-3

D1090E (R) 5-AGCCAAGCCGTCTTCGCCGTTATTGCG-3

D1091E (F) 5-AATAACGGCGACGAAGGCTTGGCTGTC-3

D1091E(R) 5-GACAGCCAAGCCTTCGTCGCCGTTATT-3

Supplementary Table S2 Kinetic Parameters

|  | *K*_m_ (mg/mL) | *k*_cat_ (sec-1) | *k*_cat_/*K*_m_ |
| --- | --- | --- | --- |
| Wild-type | 36.23 | 81.5 | 2.25 |
| D1067E | 42.14 | 6.07 | 0.144 |
| D1090E | 35.35 | 6.44 | 0.182 |
| D1091E | 72.38 | 49.7 | 0.687 |

Kinetic parameters for hydrolysis were determined by assays using 0.75–20 mg/mL α-1,3-glucan. A reaction mixture containing 0.5 nmol/mL of enzyme and 50 mm potassium phosphate buffer (pH 6.5) was incubated at 30°C. After 30 min, the reaction was stopped by boiling for 5 min. The amount of reducing sugar in the supernatant was determined. Kinetic parameters were obtained from Lineweaver–Burk plots.

Supplementary Fig. S1 Multiple alignments of amino acid sequence of catalytic domain from family 87 α-1,3-glucanases. Amino acid sequences were aligned by Clustal W. Conserved amino acids in catalytic domain of α-1,3-glucanases are indicated by a back arrow. Abbreviations and accession number of the putative α-1,3-glucanases are as follows: BcAgl-KA, α-1,3-glucanase from *B. circulans* KA-304 (BAE98302): PgAgl-FH1, α-1,3-glucanase from *P. glycanilyticus* FH11 (type I, ): PgAgl-FH2, α-1,3-glucanase from *P. glycanilyticus* FH11 (type II),: PrMut, α-1,3-glucanase from *Paenibacillus* sp. RM1 (E16590): PhMut, α-1,3-glucanase from *Paenibacillus humicus* (BAI23187): PcMutP, α-1,3-glucanase from *P.* *curdlanolyticus* (ADT91063): PkMuA, α-1,3-glucanase from *Paenibacillus* sp. KSM-35 (BAG15878): PkMuB, α-1,3-glucanase from *Paenibacillus* sp. KSM-86 (BAF56208): PkMuC1, α-1,3-glucanase from *Paenibacillus* sp. KSM-126 (type I, BAG15879): PkMuC2, α-1,3-glucanase from *Paenibacillus* sp. KSM-126 (type II, BAG15880): PkMuE, α-1,3-glucanase from *Paenibacillus* sp. KSM-138(BAH10514): PjAPHP0617, APHP domain-containing protein from *Paenibacillus* sp. JDR 2 (YP_003009383): PjAHPH3616, APHP domain-containing protein from *Paenibacillus* sp. JDR 2 (YP_003012334): PyAPHP, APHP domain-containing protein from *Paenibacillus* sp. Y412MC10 (YP_003242479) and PvAPHP, APHP domain-containing protein from *Paenibacillus* *vortex* (WP_006209693): AmHP, glycosyl hydrolase from Amycolatopsis mediterrane (WP_013226454): AmPMP, glycosyl hydrolase from *Actinoplanes missouriensis* (WP_014446698): AsAPHP, hypothetical protein from Actinoplanes sp. SE50/110 (WP_014693728): KfCB, Carbohydrate binding family 6 from *Kribbella flavida* DSM 17836 (ADB33690):SaSGH, Secreted glycosyl hydrolase from Stigmatella aurantiaca DW4/3-1 (ADO68245) :SsMyc: mycodextranase from *Streptomyces* sp. J-13-3 (BAB62749).


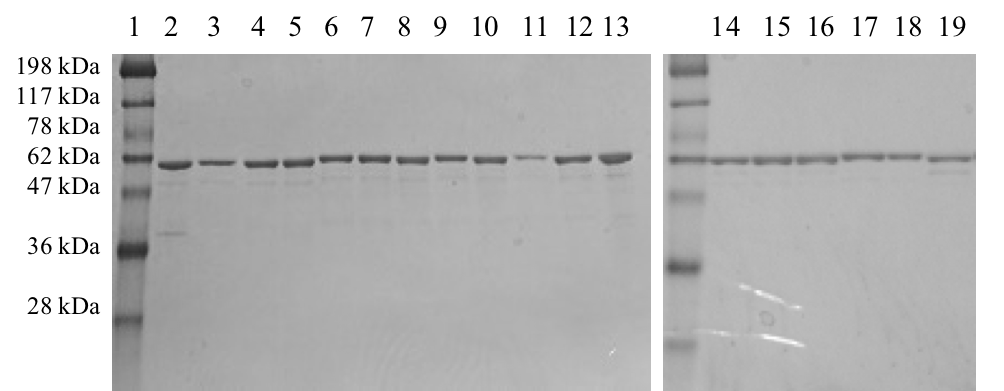


Supplementary Fig. S2 SDS-PAGE analysis of wild type enzyme and mutant enzymes. 12.5% of SDS-PAGE was used. The gel was stained with Coomassie Brilliant Blue R-250. Lane1, Marker: 2, AglΔDCD-UCD wildtype: 3, D737A: 4, D739A: 5, E763A: 6, D853A: 7, E854A: 8, D884A: 9, E889A: 10, E1032A: 11, D1067A: 12, D1090A: 13, D1091A: 14, D1067N: 15, D1090N: 16, D1091N: 17, D1067E: 18, D1090E: 19, D1091E


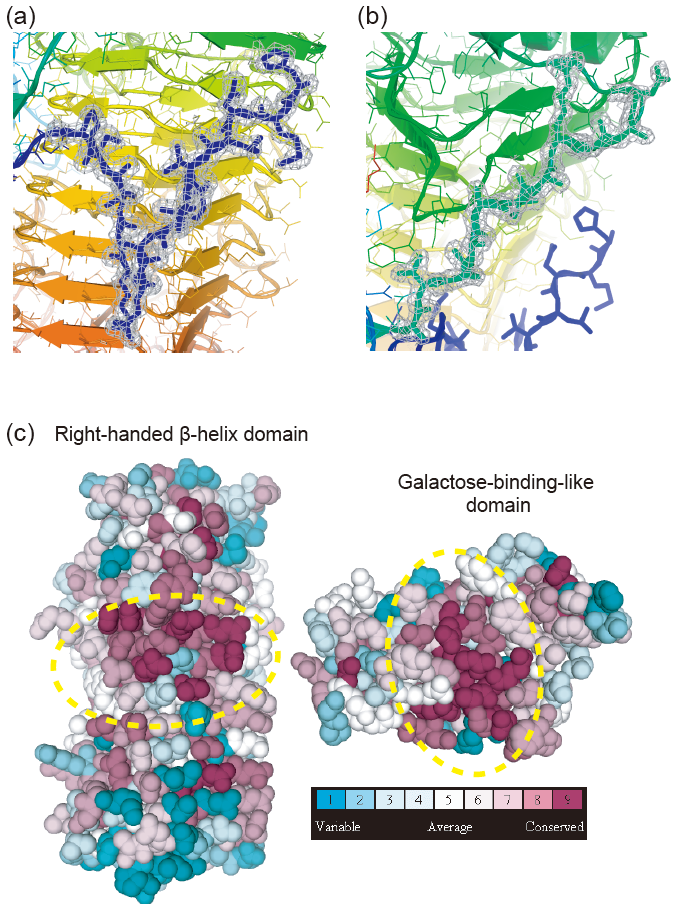


Supplementary Fig S3. (a, b) The electron density maps for the N-terminal extension (a) and the linker between the galactose-binding-like domain and the β-helix domain (b). The electron density maps are shown around the focusing residues with 1.0 σ contour level. (c) Amino acid conservation at the interface between the galactose-binding-like domain and the β-helix domain. Interface areas between the domains are shown with yellow dotted circles.


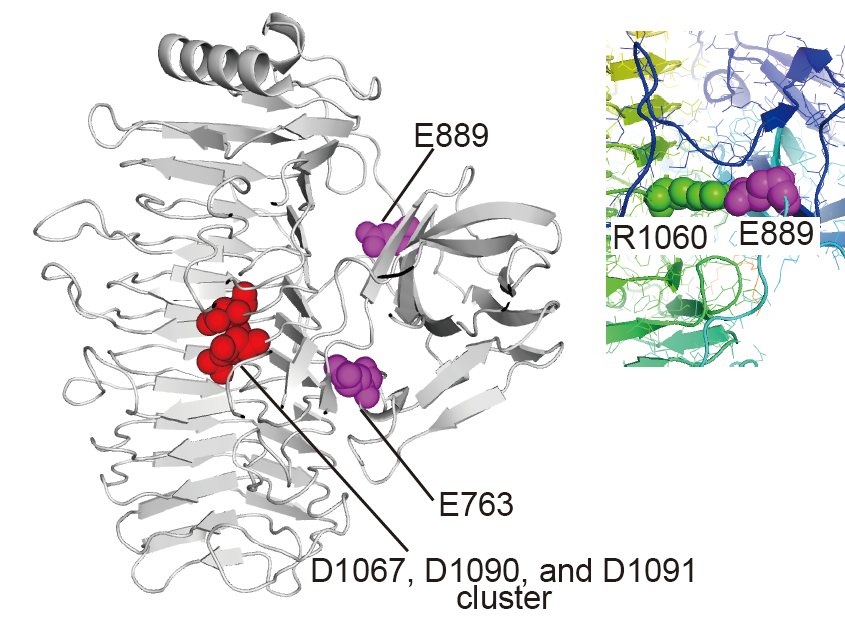


Supplementary Fig S4. (Left) Mutational sites important for the enzymatic activity revealed by the biochemical assays. E763 and E889 are shown in purple and D1067, D1090, and D1091 are shown with red CPK representations. (Right) The inter-domain ionic bonding pair between E889 and R1060.
